# Coupled Effects of Tree Species and Understory Morel on Modulating Soil Microbial Communities and Nutrient Dynamics

**DOI:** 10.3390/microorganisms14010099

**Published:** 2026-01-02

**Authors:** Xia Yuan, Haiyan Qin, Yun Wang, Shuwen Wu, Zeyu Zhang, Muxin Fan, Li Li, Liuqian Tian, Yiwen Fu

**Affiliations:** 1 School of Life and Environmental Sciences, Hangzhou Normal University, Hangzhou 311121, China; xyuan@hznu.edu.cn (X.Y.);; 2 School of Environment and Surveying Engineering, Suzhou University, Suzhou 234000, China

**Keywords:** understory intercropping, morel cultivation, soil microorganisms, nutrient dynamics, mineral elements

## Abstract

Morel mushrooms (*Morchella* spp.) are highly prized for their culinary and economic value. Understory cultivation, leveraging the symbiotic relationship between morels and trees, has gained increasing popularity. However, the effects of this practice on belowground microbial communities and nutrient dynamics remain poorly understood. In this study, we examined how understory cultivation of morels (*Morchella sextelata*) under five different tree species affects soil bacterial and fungal communities, as well as nutrient availability and mineral element content. The results revealed that soil physicochemical properties responded variably to morel cultivation under different tree species. Notably, understory morel cultivation reduced soil NO_3_^−^-N by 38–67% across tree species, whereas NH_4_^+^-N remained stable, reflecting the distinct nutrient preference of *Morchella* and associated trees, and suggesting targeted nitrate fertilization could mitigate nitrogen limitations. Understory cultivation significantly increased soil mineral elements, with *Zelkova serrata* (*Z. serrata*) showing the highest concentrations, elevating available potassium (AK), calcium (ECa), manganese (AMn) and boron (AB) by approximately 20%, 13%, 30%, and 168%, highlighting its potential for soil quality improvement. Microbial community composition was also significantly altered, with fungal communities exhibiting more pronounced shifts than bacterial communities, likely due to their closer ecological associations with morels. Importantly, *Z. serrata* markedly promoted microbial-mediated soil carbon and nitrogen accumulation, driven by mineral binding, root secretions and soil pH value. These findings enhance understanding of belowground effects of morel understory cultivation, revealing that select tree species like *Z. serrata* can improve soil quality and nutrient cycling, while targeted nitrate fertilization supports sustaining morel cultivation systems.

## 1. Introduction

Morel mushrooms (*Morchella* spp.) are highly valued edible fungi and have garnered considerable attention for their unique flavor and substantial nutritional benefits [1]. Due to rising demand and the scarcity of wild populations, the cultivation of morels has become essential for sustainable production and commercial development [2]. In recent years, understory cultivation has emerged as an eco-friendly agricultural practice. It utilizes the symbiotic relationship between trees and morels, thereby yielding both ecological and economic benefits [3]. Understanding how understory planting influences soil microbes is essential to elucidating the tree–morel–microbe interplay and supporting sustainable agroforestry and morel cultivation ecology. However, research on the intricate effects and mechanisms by which understory trees and morels shape belowground microorganisms and nutrient dynamics remains limited.

Tree roots significantly influence soil physicochemical properties through water and nutrient uptake. Their root systems impact soil surface area, improve water absorption efficiency [4], and alter soil pH and organic matter content via root exudates. As key soil inhabitants, fungi exhibit remarkable adaptability to environmental stresses and play crucial roles in organic matter decomposition and carbon-nutrient cycling [5]. Fungal diversity and activity are modulated by multiple biotic factors (e.g., plants and associated organisms) and abiotic factors (e.g., soil pH, moisture, salinity, and temperature) [6]. Of particular ecological significance in forest ecosystems is ectomycorrhizal symbiosis between trees and soil fungi [7]. These mycorrhizal fungi mediate interactions between plant and soil microbiomes, facilitate relationships with nitrogen-fixing bacteria, phosphorus-solubilizing microbes, vitamin-producing mutualists, and pathogens. Through these interactions, they further regulate belowground plant traits, influence plant-plant relationships, and modify ecosystem processes in conjunction with other environmental factors [8]. Yet, the mechanisms by which the root systems of different tree species shape the soil microenvironment are unclear, especially their effects on soil nutrients, which warrant further investigation.

The soil microbiome represents a ubiquitous and essential component of ecosystems, influencing plant development through its structural and functional characteristics [9]. Trees substantially shape microbial community structure via root exudates and litter inputs; distinct tree species foster unique microbial assemblages, reflecting species-specific selection pressures [10,11]. Notably, edible macrofungi such as morels can markedly alter soil microbial communities [12]. For instance, the mycelia of *Morel esculenta* can enhance the activities of soil invertase and amylase, thereby changing the metabolic activity of soil microorganisms. In addition, morel cultivation can increase soil bacterial abundance and diversity, suggesting its beneficial role in this regard [12,13]. Furthermore, under coexistence conditions, the effects of trees and fungi on soil microbial community structure are interactive [14]. Trees provide substrates for fungal growth through root exudates and litter, while fungal hyphal networks stimulate microbial proliferation and metabolic activity [15]. These complex interactions form ecological networks that maintain the stability and diversity of soil ecosystems. Nevertheless, existing research has largely focused on the individual effects of trees or edible fungi on soil microbes, and systematic studies on their synergistic or antagonistic interactions are still limited. This gap hinders elucidation of key drivers regulating microbial community dynamics in understory morel systems.

Understory forest cultivation is a sustainable agroforestry practice that enhances the efficiency of forest land utilization while optimizing forest resources, spatial configuration, and ecological conditions [16]. Notably, it alters soil physicochemical properties, mineral element composition, microbial activity, and environmental carbon-nitrogen dynamics. Vegetation type has been shown to significantly modify nitrogen cycling processes, though the effects vary substantially [17]. Understory morels may reduce soil nitrogen availability by competing with plants for nitrogen or consuming labile carbon that would otherwise support ammonifying microorganisms, further limiting nitrification potential [18]. Moreover, morels form symbiotic relationships with plants, enhancing water and nutrient uptake through extensive mycelial networks that permeate soil and nutrient layers [19]. These networks facilitate bidirectional nutrient transport between resource-rich and resource-poor zones [20]. Meanwhile, root mechanics and the availability of carbon substrate foster the emergence of specific microbial communities in understory soil [21]. Overall, much of the research on understory planting has focused on the growth of the plants themselves or changes in microbial community. However, there is limited research on the symbiotic relationship between trees, understory plants, and changes in soil microorganisms within ecosystems.

Among various species, *Morchella sextelata* stands out as the primary candidate for artificial domestication [22]. To clarify the carbon and nitrogen fluxes, soil properties, and microbial dynamics in understory morel cultivation systems, this study focuses on *M. sextelata* morel cultivation under different tree species. Employing high-throughput sequencing and physiological-biochemical analyses, we examine how morel cultivation influences soil microbiota and elucidate the underlying mechanisms. The aim is to provide a theoretical basis and technical support for morel cultivation under diverse trees to promote sustainable agricultural development. Three key research questions are addressed: (1) How do trees and morels interact to partition soil nitrogen resources? (2) How do morels regulate soil microbial diversity, and do they exert divergent effects on bacterial versus fungal communities? (3) Can the selectivity of different tree species for soil mineral elements and microbial communities facilitate soil carbon and nitrogen accumulation?

## 2. Materials and Methods

### 2.1. Experimental Design

To evaluate soil impacts of different understory morel cultivation systems, we selected five prevalent roadside afforestation tree species at the North Anhui Comprehensive Experimental Station (33°41′15.36′′ N, 117°05′32.76′′ E): *Begonia evansiana*, *Zelkova serrata* (*Thunb.*) *Makino*, *Koelreuteria paniculata Laxm.*, *Ligustrum lucidum Ait.* and *Acer palmatum* ‘*Atropurpureum*’. We established three replicate experimental greenhouses (each 44 m × 3 m × 1.8 m) in the understory of each tree species, along with three control greenhouses in nearby open areas without trees, totaling 18 greenhouses spaced 4 m apart. All greenhouses were specifically designed for cultivating *Morchella sextelata*.

In December 2022, prior to sowing *M. sextelata*, the soil in each greenhouse was uniformly tilled to a depth of 15–20 cm using an Dongfanghong LY1004(G4) agricultural four-wheel rotary tiller to standardize growing conditions (China YTO Group Corporation, Luoyang, China). The soil was classified as Calcic Vertisol (World Reference Base; IUSS Working Group WRB 2015). *M. sextelata* spawn mixed with cultivation medium (78% wheat, 20% corn cob, 1% lime, 1% gypsum) was mechanically sown at 0.3 kg m^−2^ using a morel seeder to ensure uniform distribution (2B XF-24, Shandong Qichen Machinery Manufacturing Factory, Weifang, China). Seven to ten days post-sowing, nutrient bags (1 kg each; 50% wheat, 48% corn cob, 1% lime, 1% gypsum) were added at a density of 3 bags m^−2^. To maintain soil moisture at 17–35%, air humidity at 85%, and temperatures below 20 °C, all greenhouses are covered with perforated black polyethylene film. Ventilation is adjusted to keep CO_2_ concentration below 2000 ppm, which allows for precise control over environmental conditions. Morel fruiting bodies were harvested in mid-March 2023, and fresh weight per greenhouse was recorded to calculate yield (kg m^−2^).

### 2.2. Soil Sampling

After the morel harvest, five 1 m × 1 m sampling plots were randomly set up in each greenhouse following an ‘S’-shaped pattern. Adjacent plots were spaced at least 2 m apart and all plots were kept more than 0.5 m away from the greenhouse edges. Soil samples from each plot were collected to a depth of 10 cm [23]. Samples collected from the same greenhouse were combined into a composite sample, resulting in a total of 18 soil samples (6 treatments × 3 replicates). The samples were promptly transported to the laboratory in a refrigerated container, where impurities (e.g., stones, roots) were removed. The samples were then divided into three parts for different analysis. The first part was air-dried and used to determine soil carbon and nitrogen content, pH, and mineral elements. The second part was refrigerated at 4 °C for the measurement of soil water content (SWC), electrical conductivity (EC), available phosphorus (AP), soil available nitrogen (NH_4_^+^-N and NO_3_^−^-N), and enzyme activity. The third part was stored at −80 °C for DNA extraction.

### 2.3. Soil Physicochemical Analysis

Soil pH was determined using a multi-parameter water quality analyzer (DZS-706F-Af-A, Shanghai Leici Instrument Co., Shanghai, China) in a 1:2.5 soil-to-deionized water suspension. SWC was measured gravimetrically by oven-drying samples at 105 °C until a constant weight [8]. For the analysis of SOC and TN, samples were first acidified with 1 M HCl to remove carbonates, followed by analysis using an elemental analyzer (Vario EL III, Elementar, Germany). NO_3_^−^-N were assayed using UV-spectrophotometry (UV-8000, Metash, Shanghai, China), and NH_4_^+^-N via the indophenol blue method [23]. AP was determined using the Olsen method [24]. Available potassium (AK) was extracted with neutral ammonium acetate and quantified by flame photometry. Available iron (AFe) and available manganese (AMn) were extracted with DTPA and determined by atomic absorption spectrophotometry [25]. Available boron (AB) was quantified via the hot water extraction method coupled with spectrophotometry. Exchangeable calcium (ECa) and exchangeable sodium (ENa) were extracted with neutral ammonium acetate and analyzed by atomic absorption spectrophotometry [26].

### 2.4. The Determination of Soil Enzyme Activities

The activities of three soil hydrolases were measured using a 96-well microplate fluorescence assay [27]. The enzymes measured were variable β-glucosidase (BG), which is associated with the carbon cycle; N-acetyl-β-D-glucosidase (NAG), which is associated with the nitrogen cycle; and acid phosphatase (AcP), which is associated with the phosphorus cycle. Briefly, 1.5 g of each soil sample was weighed into 150 mL Tris-HCl buffer solution (pH 7.5), and the mixture was thoroughly stirred for 15 min. The suspension and the corresponding reagents were then added to the appropriate wells of a black 96-well microplate (eight technical replicates), bringing the final volume to 250 μL per well. The plate was then incubated at 25 °C in the dark for three hours. Finally, we measured the quantity of fluorescence at an excitation wavelength of 360 nm and an emission wavelength of 460 nm using a microplate reader (Synergy H1M, BioTek, Shoreline, WA, USA). The units for hydrolytic enzyme activities were nmol g^−1^ dry soil h^−1^.

### 2.5. High-Throughput Sequencing

Soil microbial genomic DNA was extracted using the ALFA-SEQ Advanced Soil DNA Kit (mCHIP BioTech Co., Ltd., Guangzhou, China) following manufacturer’s protocols. Bacterial and fungal communities were analyzed via Illumina MiSeq sequencing (Illumina, San Diego, CA, USA). Bacterial 16S rRNA genes were amplified using the primers 515F (5′-GTGCCAGCMGCCGCGG-3′) and 806R (5′-CCGTCAATTCMTTTRAGTTT-3′) [28]. The PCR program was as follows: initial denaturation at 94 °C for 5 min; 32 cycles of 94 °C for 30 s, 53 °C for 30 s, and 72 °C for 30 s; and a final extension at 72 °C for 8 min. For fungi, the ITS1 regions were amplified with the primers BD-ITSIF (5′-GGAAGTAAAAGTCGTAACAAGG-3′) and ITS2-2043R (5′-GCTGCGTTCTTCATCGATGC-3′). The PCR conditions included an initial denaturation at 95 °C for 3 min, followed by 34 cycles of denaturation at 95 °C for 20 s, annealing at 56 °C for 20 s, and extension at 72 °C for 30 s; and a subsequent final extension at 72 °C for 5 min. All PCR reactions had a total volume of 25 μL, containing 12.5 μL × Taq Master Mix, 1 μL of each primer (10 μM), and 10.5 μL of DNA template.

Sequence data processing was conducted using Quantitative Insights Into Microbial Ecology 2 (QIIME 2) for quality filtering, error correction, and preliminary taxonomic assignment. Bacterial and fungal reads were denoised with the Divisive Amplicon Denoising Algorithm (DADA 2) [29], which was used for denoising, filtering, merging, and chimera removal, as well as generating amplicon sequence variants (ASVs). Taxonomic identification of ASVs was performed using the feature-classifier plugin in QIIME 2 [30], which aligned the representative sequences of ASVs to the SILVA database (16S rRNA) and the UNITE database (ITS) with a 99% similarity threshold. Classification confidence was assessed using the classify-sklearn naïve Bayes taxonomy classifier with default parameters.

### 2.6. Statistical Analyses

All statistical analyses were conducted using R 4.4.2. The effects of different treatments on morel yield, soil properties, enzyme activities, and microbial variables were assessed using one-way analysis of variance (ANOVA) combined with Tukey’s test (*p* < 0.05). Equality of variances was verified prior to conducting the ANOVA. Microbial β-diversity was evaluated through principal coordinates analysis (PCoA) based on Bray–Curtis dissimilarity matrices. The significance of differences in microbial β-diversity among groups was determined using permutational multivariate analysis of variance (PERMANOVA) run with 999 permutations (*n* = 3) [31]. Taxonomic biomarkers were identified using linear discriminant analysis effect size (LEfSe) with an LDA score threshold of >3.0 (log_10_, *p* < 0.05). Additionally, the random forest classification method was employed via the Random-Forest package to predict the key fungal phyla within six grouped categories (estimators = 500) [32]. Moreover, the associations between soil physicochemical properties and soil microbes were evaluated using the Mantel test and Pearson correlation analysis with ggcor R package (v 0.9.7). The probability level of 0.05 was considered statistically significant.

## 3. Results

### 3.1. The Yield of Morels

**Figure 1:**
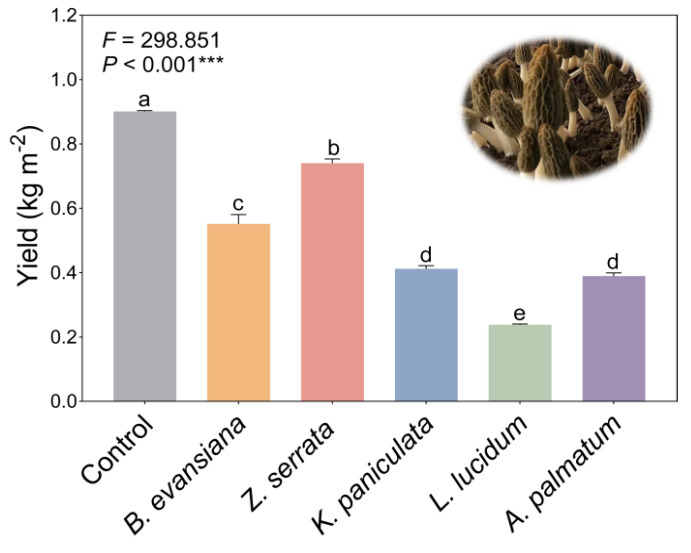
Yield of morels grown under different forests. Each value and bar are repeated mean values and ±SE (*n* = 3). According to one-way ANOVA analysis, values sharing a common letter are not significant (*p* < 0.001). The *p* values are expressed as follows: *** *p* < 0.001.

Morel yields varied significantly among different understory planting systems (*p* < 0.001), with all tree species showing suppressive effects compared to open-field cultivation (Figure 1). One-way ANOVA revealed that the negative impact on morel yield was most pronounced in the *L. lucidum* understory, followed by *K. paniculata* and *B. evansiana* understories, while the suppressive effect was weakest in the *Z. serrata* understory.

### 3.2. Physico-Chemical Properties of Soil

**Figure 2:**
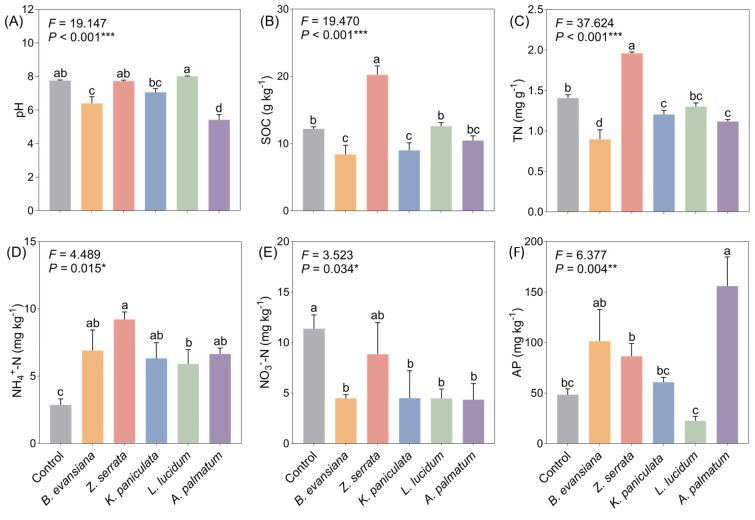
Different responses of soil properties to understory morel cultivation. (**A**) pH; (**B**) SOC, soil organic carbon; (**C**) TN, total N; (**D**) NH_4_^+^-N, ammonium N; (**E**) NO_3_^−^-N, nitrate N; (**F**) AP, Available P. Each value and bar are repeated mean values and ±SE (*n* = 3). According to one-way ANOVA analysis, values with the same letter are not significant (*p* < 0.05). The *p* values are expressed as follows: * *p* < 0.05; ** *p* < 0.01; *** *p* < 0.001.

Comparative analysis revealed distinct impacts of forest versus non-forest cultivation on soil physicochemical properties (Figure 2). Most soil quality indices were significantly lower under trees, except for NH_4_^+^-N and AP. Although no significant differences were found in soil pH and NO_3_^−^-N levels between *Z. serrata* understory and open-field cultivation (Figure 2A,E), *Z. serrata* understory soils exhibited higher SOC and TN contents (Figure 2B,C, *p* < 0.001). Generally, understory soils had higher NH_4_^+^-N but lower NO_3_^−^-N than the control (Figure 2D,E). AP content was elevated in understory soils except *L. lucidum* (Figure 2F, *p* = 0.004). Overall, soil nutrients responded differentially to understory morel cultivation, with NO_3_^−^-N and NH_4_^+^-N showing contrasting patterns, while *Z. serrata* exhibited a distinct advantage in carbon and nitrogen sequestration.

### 3.3. Soil Mineral Elements

**Figure 3:**
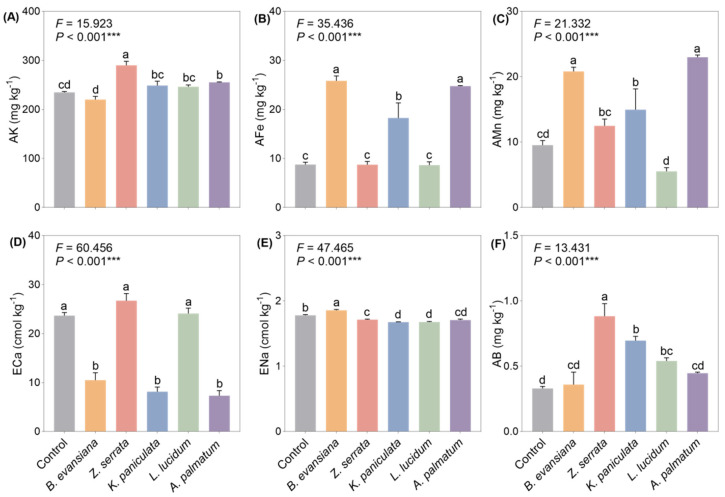
Different responses of soil mineral elements to understory morel cultivation. (**A**) AK, available K; (**B**) AFe, available Fe; (**C**) AMn, available Mn; (**D**) ECa, exchangeable Ca; (**E**) ENa, exchangeable Na; (**F**) AB, available B; Each value and bar are repeated means values and ±SE (*n* = 3). According to one-way ANOVA analysis, values with the same letter are not significant (*p* < 0.05). The *p* values are expressed as follows: *** *p* < 0.001.

Compared to the control, the understory soils beneath different tree species showed higher or comparable levels of mineral elements (AK, AFe, AMn, and AB) relative to non-understory soils, whereas ECa and ENa concentrations were generally lower (Figure 3). In general, planting morel under *Z. serrata* increased soil AK and AB contents while decreasing ENa, with no significant changes in AFe, AMn, or ECa (Figure 3A–F). Specially, AFe content was significantly reduced under *K. lucidum*, *L. paniculata,* and *B. evansiana* compared to non-understory soils, while *Z. serrata* had no significant effect on AFe content (Figure 3B). In contrast, AMn content was significantly elevated in all understory soils except *L. lucidum*, with the highest levels in *A. palmatum* and *B. evansiana* (Figure 3C). A similar trend was observed under *L. lucidum* and *Z. serrata*, though AMn levels were lower than in non-forested plantations. Therefore, understory morel cultivation increased the levels of most mineral elements but reduced the levels of soluble salts (ECa and ENa), with the effect being most significant in *Z. serrata*.

### 3.4. Soil Enzyme Activities

**Figure 4:**
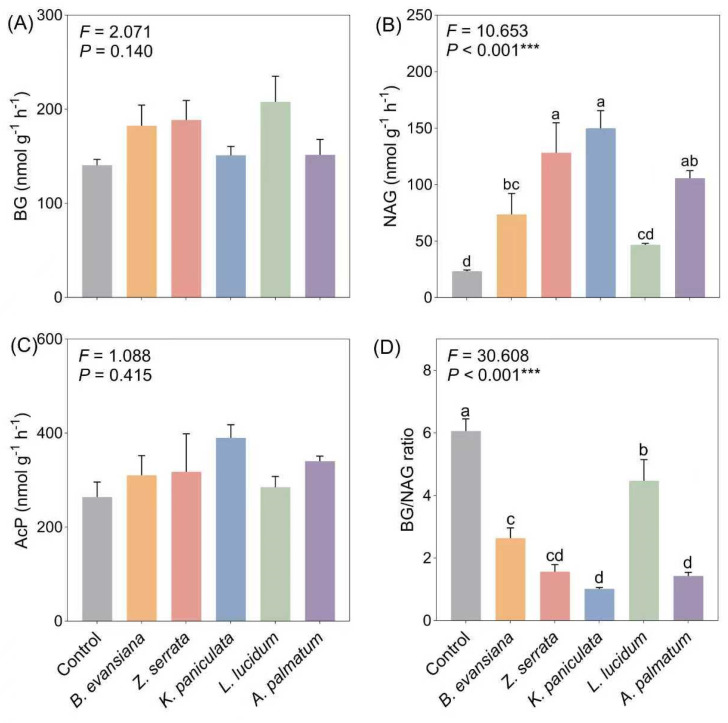
Different responses of soil enzyme activities and their chemical ratios to understory morel cultivation. (**A**) BG, β-1,4-glucosidase; (**B**) NAG, 1,4-N-acetylglucosaminidase; (**C**) AcP, acid phosphatase; (**D**) BG/NAG ratio. Each value and bar are repeated mean values and ±SE (*n* = 3). According to one-way ANOVA analysis, values with the same letter are not significant (*p* < 0.05). The *p* values are expressed as follows: *** *p* < 0.001.

The value of soil enzyme activities in understory morels was generally higher than those in non-understory morels (Figure 4). Although no statistical significance was observed across tree treatments, BG activity under L. lucidum was the highest, 66.37% higher than the control (Figure 4A). Similarly, NAG activity was obviously higher across all understory morels, with the highest levels observed under *K. paniculata* (Figure 4B, *p* < 0.001). It is worth noting that the BG/NAG ratio was opposite to the soil enzyme activity, and the BG/NAG ratio of non-forest morel was higher than the forest, of which *L. lucidum* grove had the highest BG/NAG ratio, and *K. paniculata* grove had the lowest BG/NAG ratio (*p* < 0.001). Compared with the control group, the cultivation of Understory morel has a significant advantage in promoting microbial enzyme activity.

### 3.5. Soil Microbial Diversity

**Figure 5:**
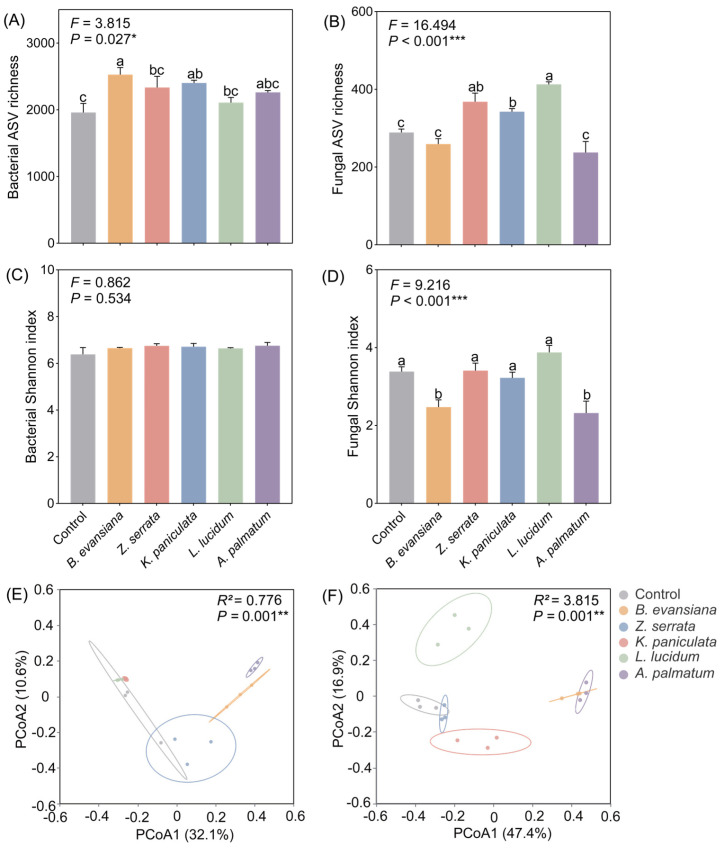
Different responses of soil microbial diversity to understory morel cultivation. Richness index of bacteria (**A**) and fungi (**B**). Shannon index of bacteria (**C**) and fungi (**D**). β diversity of bacterial community (**E**) and fungal community (**F**) by PCoA analysis based on Bray–Curtis distance. PERMANOVA was employed to examine differences among various soil microbial communities. Each value and bar were repeated mean values and ±SE (*n* = 3). According to one-way ANOVA analysis, values sharing a common letter are not significant (*p* < 0.05). The *p* values are expressed as follows: * *p* < 0.05, ** *p* < 0.01, *** *p* < 0.001.

The alpha diversity (richness index and Shannon-Wiener index) of soil bacterial and fungal communities across different understory morel plantings was assessed using amplicon sequence variants (ASVs) (Figure 5A–D). Compared with the control, understory morel cultivation significantly influenced both fungal alpha diversity (Figure 5B,D, *p* < 0.001). For bacterial communities, only the richness index showed significant differences (*p* = 0.027), with the treated groups generally exhibited higher richness than the control, especially under *B. evansiana* and *K. paniculate* treatments. For fungal communities, the richness and Shannon-Wiener indices in the *Z. serrata*, *K. paniculata*, and *L. lucidum* were significantly higher than those in the control (*p* < 0.001). These results demonstrate that *Z. serrata*-morel association enhanced microbial diversity, particularly among fungi.

Beta diversity, assessed via Bray–Curtis-based PCoA for both bacterial (Figure 5E) and fungal communities (Figure 5F), revealed distinct microbial community structures. PCoA1 accounting for 32.1% and PCoA2 for 10.6% of the total variance in bacterial communities. Fungal communities showed greater separation, with PCoA1 accounting for 47.4% and PCoA2 for 16.9% of the total variance. The control and *Z. serrata* treatment groups were close to each other and independent of the fungal communities in the other treatment groups (*p* = 0.001, Figure 5F).

### 3.6. Soil Microbial Community Composition

**Figure 6:**
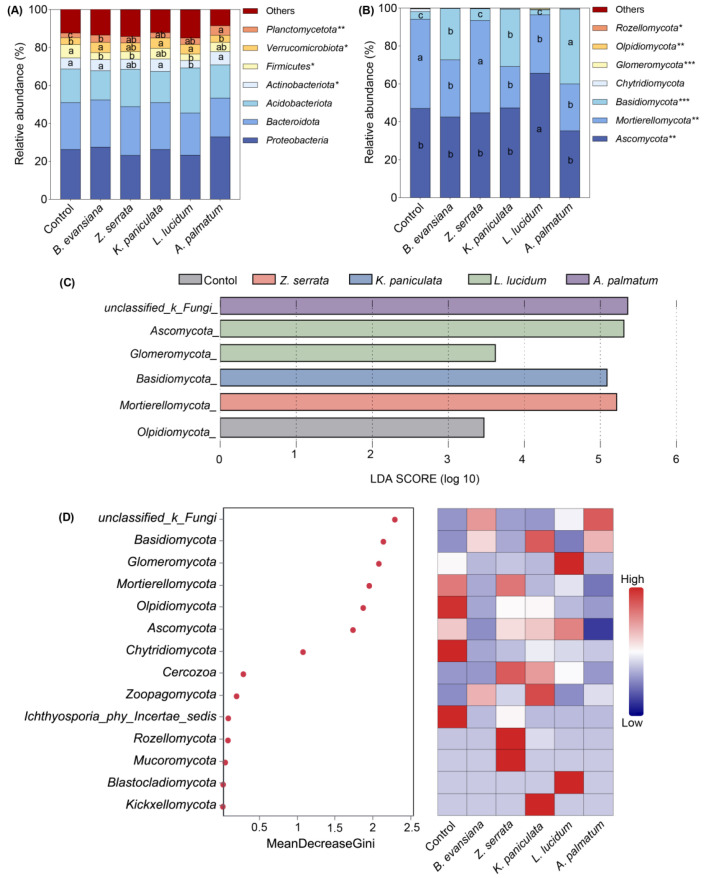
Relative abundance of bacterial and fungal phyla in soil samples. The seven most dominant bacterial (**A**) and fungal (**B**) phyla in soil samples; LEfSe analysis of six fungal communities in different forests (**C**); Random forest modeling with correlation heatmap (**D**). The lowercase letters (a–c) in the stacked bar chart indicate significant differences at the 0.05 level between the different bacterial (A) and fungal (B) categories (n = 3). P-values are denoted as follows: * *p* < 0.05; ** *p* < 0.01; *** *p* < 0.001. The random forest model achieved a 14-fold cross-validation accuracy of 54.26%.

The composition of bacterial and fungal communities at the phylum level is presented in Figure 6A,B. Among soil samples from each treatment group, the relative abundance of *Mortierellomycota* in morel cultivar under *Z. serrata* (*p* = 0.01), which had the highest yield, was significantly higher than that in the control group and the other treatment groups. Conversely, the relative abundance of *Ascomycota* was slightly higher in *L. lucidum* understory morels (the lowest-yielding group) compared to the control and other groups (*p* = 0.006). These findings suggest a potential association between fungal community composition, morel yield, and tree species.

Linear discriminant analysis effect size (LEfSe) and random forest modeling were employed to identify predictive fungal taxa in morel-cultivated soils across different tree species. LEfSe analysis revealed that the most prominent fungal genera corresponded to those with the highest relative abundance in morel-cultivated soils under each tree species. Using a random forest approach, 13 key fungal genera were identified as statistically significant predictors of distinct community composition patterns. Specifically, *Glomeromycota* showed high predictability for *L. lucidum* understory cultivation, while *Olpidiomycota* and *Chytridiomycota* communities were highly predictive of non-understory *L. lucidum* cultivation. Additionally, *Ascomycota* had a high negative predictive value for morel cultivation under *A. palmatum*.

### 3.7. Regulatory Factors Influencing Soil Microbes

**Figure 7:**
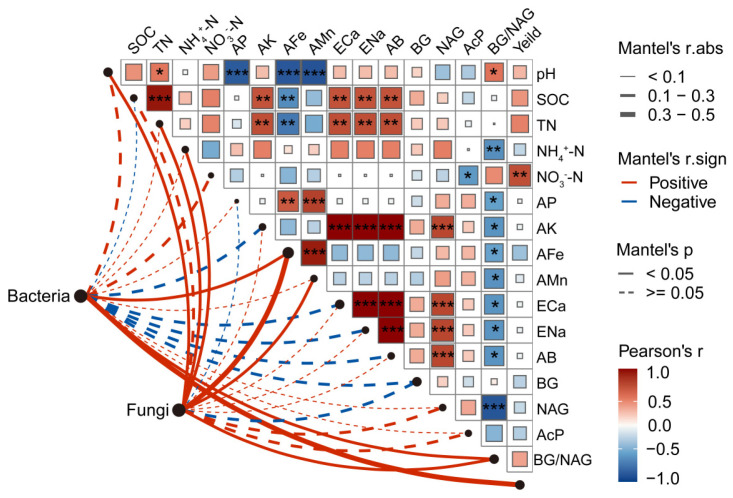
Correlation between soil enzyme activities, soil physicochemical characteristics, mineral elements and microbial community composition in different understory morel cultivated soils. Edge width presented the mantels r statistic with the corresponding width. Color gradient presented Pearson’s r correlation coefficient. Line width and the color correspond to the Mantel’s *r* and *p* statistic. SOC, soil organic carbon; TN, total N; NH_4_^+^-N, ammonium N; NO_3_^−^-N, nitrate N; AP, available P, AK, available K; AFe, available Fe; AMn, available Mn; ECa, exchangeable Ca; ENa, exchangeable Na; AB, available B, BG, β-1,4-glucosidase; NAG, 1,4-N-acetylglucosaminidase; AcP, Acid phosphatase. P-values are denoted as follows: * *p* < 0.05; ** *p* < 0.01; *** *p* < 0.001.

Mantel test and correlation analysis revealed stronger associations between fungal communities and soil parameters (pH, TN, NH_4_^+^-N, AFe, and AMn) compared to bacterial communities (Figure 7). Specifically, fungal communities showed significant positive correlations with soil pH, TN, NH_4_^+^-N, AFe, AMn, and morel yield. Notably, morel yield was only significantly and positively correlated with NO_3_^−^-N, while no significant correlations were observed with other indicators. Furthermore, significant correlations were observed between soil carbon, nitrogen, and mineral elements, with AFe and AMn exhibiting a distinct negative relationship, while all others were positively correlated.

## 4. Discussion

### 4.1. Different Responses of Soil Nutrients and Mineral Elements to Understory Morel Cultivation

Soil physicochemical properties showed significant responses to understory morel cultivation, with nitrogen (N) dynamics being particularly pronounced. Specifically, the concentration of NO_3_^−^-N in understory soil decreased significantly compared to the control group, while NH_4_^+^-N increased markedly. These findings are consistent with those of existing meta-analyses, which suggest that forest plants preferentially absorb NO_3_^−^-N, leading to the retention of NH_4_^+^-N [33]. This divergence demonstrated that tree-morel coexistence promotes competitive N partitioning through differential resource utilization strategies [34]. We propose that the preferential uptake of NO_3_^−^-N by plants is one contributing factor. Additionally, morel mycelia not only accelerate organic N mineralization, thereby increasing total available N, but also compete tree roots for the NO_3_^−^-N pools, leaving NH_4_^+^-N accumulates in the soil [35]. Such complementary N acquisition pathways represent a classic niche-partitioning strategy that reduces interspecific competition through resource specialization [36]. Crucially, the strong positive correlation between morel yield and residual NO_3_^−^-N implies that nitrate nitrogen could be a primary constraint on morel productivity in forest understories, highlighting a direct trade-off between tree and microfungal N demands.

Plants play important roles in regulating soil mineral cycling, and responses can vary between species [37]. This study revealed that understory morel cultivation significantly altered the availability of measured mineral elements. It is noteworthy that the effects and mechanisms of interplanting morels with different tree species on various minerals are distinct. For instance, elevated H^+^ concentrations promote the dissolution of Fe-Mn oxides, consequently increasing the concentrations of AFe and AMn [38]. This is supported by the significant negative correlations observed between soil pH and metal micronutrients. This process also aligns with the modification of the rhizosphere environment via root exudates (e.g., organic acids) as reported by Salas-Gonzalez et al. [39]. In contrast, AK, ECa, ENa, and AB exhibited strong positive correlations with SOC and TN, highlighting organic matter’s role in shaping the dynamics of these elements. This pattern can be explained by several interrelated mechanisms: (1) Decomposition of SOM releases potassium in plant-available K while reducing its fixation [40]; (2) Dissolved organic matter (DOM) as a byproduct of SOM breakdown could enhance boron solubility [41]; (3) SOM also influences ECa and ENa dynamics by modulating cation exchange capacity [42]. Notably, AK, ECa, and AB concentrations were peaked in the *Z. serrata*-morel interplanting system, implying that this specific plant-fungus combination may more effectively accelerate soil organic matter turnover, thereby enhancing the release of these minerals. Since the litter layer did not directly affect the soil in the greenhouse, the influence of tree roots was prioritized in this study. Further investigation of the impact of litterfall is necessary to comprehensively understand the nutrient cycling mechanisms within the forest-based morel mushroom system.

### 4.2. Diversified Responses of Soil Microbial Communities to Understory Morel Cultivation

This study revealed that understory cultivation of *Morchella* significantly altered both bacterial and fungal diversity in the soil, but their responses differed. Overall, bacterial diversity increased consistently under understory cultivation, likely driven by elevated AFe and AMn levels associated with decreased soil pH. The positive correlations among AFe, AMn and bacterial richness supported this relationship (Figure S1). Meanwhile, understory soils with higher NH_4_^+^-N content had significantly higher bacterial diversity than the control group. These findings are consistent with existing literature, which indicates that in acidic soils, bacterial communities tend to utilize more stable NH_4_^+^-N [43]. In contrast, fungal diversity exhibited a significant negative correlation with AFe and AMn, while increasing notably under specific tree species, including *Zelkova serrata*, *Koelreuteria paniculata*, and *Ligustrum lucidum*. This pattern suggests fungal communities are either competed by bacteria for resources or suppressed under high metal availability. Moreover, the rise in AP further constrained fungal diversity, consistent with documented inhibitory effects of excessive P on mycorrhiza formation [44].

Fungal communities showed more pronounced compositional shifts than bacterial communities, as evidenced by both PCoA ordination and changes in dominant phyla. Notably, soil fungal assemblages showed high similarity between control and *Z. serrata* groups. These two groups, *Mortierellomycota* and *Olpidiomycota*, consist primarily of oligotrophic fungi characterized by low carbon requirements and rapid growth rates [45]. Particularly, *Mortierellomycota* had significantly higher relative abundance in both groups and showed strong association. This phylum contributes to nutrient cycling by decomposing complex organic matter into dissolved organic carbon (DOC) and nitrogen (NH_4_^+^-N and NO_3_^−^-N). Additionally, the bacterial community in this study exhibited positive effects on morel yield, potentially due to interactions between morel and specific bacterial phyla [46]. This is highlighted by the dominance of *Firmicutes* in the control group, which is more than twice as abundant as in the other groups. Previous studies also proved that *Firmicutes* can promote morel growth and yield by inhibiting pathogenic fungi [12].

Furthermore, our results revealed that understory morel cultivation induced significant enhancement of soil NAG activity, establishing NAG as the dominant responsive extracellular hydrolase during understory morel cultivation. These collective findings provide robust address our second questions concerning the soil microbial community.

### 4.3. Mechanisms of Carbon and Nitrogen Accumulation Under Conditions of Coexistence of Morels and Trees

SOC and TN dynamics varied significantly among understory soils, with *Z. serrata* showing a pronounced promotive effect. Our analysis revealed that several mineral elements play crucial roles in SOC and TN accumulation through distinct mechanisms.

Firstly, positive correlations were observed between SOC and TN accumulation and key mineral elements (AK, ECa and AB), a finding further supported by the higher retention of AK, ECa, and AB in *Z. serrata* soils. Previous studies have established that K^+^ and Ca^2+^ mitigate soil acidification, enhance soil aggregate formation, and maintain soil environmental stability [47]. Soil aggregates physically protect SOC from microbial decomposition, while a stable soil pH optimizes the activity of hydrolytic enzymes (e.g., β-glucosidase, acid phosphatase) involved in organic matter transformation. This is consistent with the higher enzyme activities detected in *Z. serrata* understory soils. Additionally, AB promotes cell wall polysaccharide synthesis in plant roots [48], enhancing soil aggregate stability and reducing SOC leaching.

Secondly, understory soil exhibited lower ENa content than the control, leading to a reduced exchangeable sodium percentage (ESP). This condition minimizes soil colloid dispersion, favors soil aggregate formation, improves microbial carbon use efficiency, and facilitates SOC and TN accumulation [49,50]. Collectively, these results indicate that while understory morel cultivation does not increase yield, it enhances soil quality by promoting carbon and nitrogen sequestration and stabilizing microbial communities.

Conversely, AFe and AMn showed negative correlations with SOC and TN, particularly AFe, which can be primarily attributed to two mechanisms. On the one hand, *Z. serrata* preferentially absorbed iron and manganese [51], resulting in significantly lower AFe and AMn levels compared to other groups. On the other hand, as an essential trace element, iron supports microbial metabolism via redox-coupled energy generation [52], and bacterial or fungal communities showed positive correlations with AFe. This suggests that iron (and manganese) could enhance the input of plant-derived organic matter input, and subsequent microbial degradation and utilization, thereby facilitating C and N accumulation in soils. Together, these findings clarify that *Z. serrata* enhances soil carbon and nitrogen accumulation by selectively regulating mineral elements availability and microbial community dynamics.

## 5. Conclusions

This study systematically explored the coupled effects of understory morel cultivation on soil ecosystems. Results revealed that nitrogen dynamics shifted significantly in understory plantations, characterized by decreased NO_3_^−^-N but increased NH_4_^+^-N levels, reflecting the distinct nutrient preferences of morels and trees, and suggesting that targeted nitrate fertilization could mitigate N limitations. Soil mineral elements exhibited varied responses, with AFe and AMn increasing with declining soil pH, while AK, ECa, and AB were closely linked to organic matter dynamics. Notably, the *Z. serrata*-morel system proved most effective in enhancing these minerals (AK, ECa, and AB), highlighting its superiority in improving soil mineral status. Understory morel cultivation significantly altered soil microbial communities, with fungal communities undergoing more pronounced shifts than bacteria. Bacterial diversity increased in relation to higher available iron and manganese and lower pH, whereas fungal diversity varied by tree species and was suppressed by excess P and elevated metals. Notably, *Mortierellomycota* (linked to C and N cycling) was most abundant in *Z. serrata* groups, likely promoting of soil carbon and nitrogen accumulation via mineral binding, root secretions and pH modulation. These findings enhance understanding of belowground effects of morel understory cultivation, revealing that select tree species like *Z. serrata* can improve soil quality and nutrient cycling, while targeted nitrate fertilization supports sustain morel cultivation system.

## Data Availability

The sequencing data have been deposited in the NCBI Sequence Read Archive (SRA) (https://www.ncbi.nlm.nih.gov/sra, accessed on 25 December 2026) under the BioProject accession number PRJNA1393779.

## Supplementary Materials

The following supporting information can be downloaded at: https://www.mdpi.com/article/10.3390/microorganisms14010099/s1, Figure S1. Spearman correlation matrix of soil physicochemical properties, extracellular enzymatic activities, and microbial diversity in different understory morel-cultivated soils. Abbreviations: SOC, Soil organic carbon; TN, Total nitrogen; NO_3_^−^-N: Nitrate nitrogen; NH_4_^+^-N, Ammonium nitrogen; AP, Available phosphorus; AK, Available potassium; AFe, Available iron; AMn, Available manganese; ECa, Exchangeable calcium; ENa, Exchangeable sodium; AB, Available boron; BG, β-1,4-glucosidase; NAG, 1,4-N-acetylglucosaminidase; AcP, Acid phosphatase. Blue indicates positive correlation and red indicates negative correlation. Statistical significance is denoted as follows: * *p* < 0.05; ** *p* < 0.01; *** *p* < 0.001.
