# Supplementary material for: Coupled Effects of Tree Species and Understory Morel on Modulating Soil Microbial Communities and Nutrient Dynamics
Source: Microorganisms. 2026 Jan 2;14(1):99. doi: 10.3390/microorganisms14010099 (PMC12844048; doi:10.3390/microorganisms14010099)
Supplement: Supplementary file 1 [file microorganisms-14-00099-s001.zip › microorganisms-4049700-supplementary.pdf]

Coupled Effects of Tree Species and Understory Morel on Modulating Soil Microbial Communities and Nutrient Dynamics

Xia Yuan [1,†], Haiyan Qin [1,†], Yun Wang [1], Shuwen Wu [1], Zeyu Zhang [1], Muxin Fan [1], Li Li [1], Liuqian Tian [1] and Yiwen Fu [2,*]

[1] School of Life and Environmental Sciences, Hangzhou Normal University, Hangzhou 311121, China; xyuan@hznu.edu.cn (X.Y.); qhy10427@163.com (H.Q.); 2024112010030@stu.hznu.edu.cn (Y.W.); 18258292210@163.com (S.W.); zhangzeyu33@163.com (Z.Z.); 13133392524@163.com (M.F.); lili1@stu.hznu.edu.cn (L.L.); 2023210301100@stu.hznu.edu.cn (L.T.)

[2] School of Environment and Surveying Engineering, Suzhou University, Suzhou 234000, China

* Correspondence: fuyiwen@caas.cn

† These authors contributed equally to this work.

Supplementary Materials

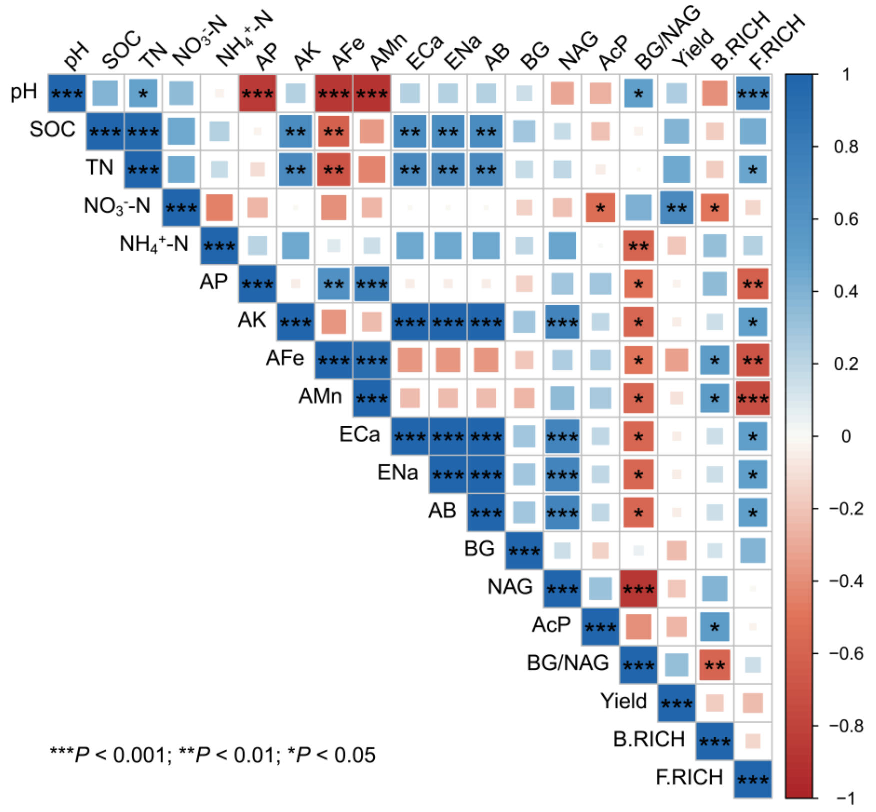

Figure S1 Spearman correlation matrix of soil physicochemical properties, extracellular enzymatic activities, and microbial diversity in different understory morel-cultivated soils. Abbreviations: SOC, Soil organic carbon; TN, Total nitrogen; NO_3^--N: Nitrate nitrogen; NH_4^+-N, Ammonium nitrogen; AP, Available phosphorus; AK, Available potassium; AFe, Available iron; AMn, Available manganese; ECa, Exchangeable calcium; ENa, Exchangeable sodium; AB, Available boron; BG, β-1,4-glucosidase; NAG, 1,4-N-acetylglucosaminidase; AcP, Acid phosphatase. Blue indicates positive correlation and red indicates negative correlation. Statistical significance is denoted as follows: * $p < 0.05$; ** $p < 0.01$; *** $p < 0.001$.
